# Tislelizumab plus chemotherapy in metastatic extramammary Paget disease after surgery: a case report

**DOI:** 10.3389/fonc.2024.1402490

**Published:** 2024-10-24

**Authors:** Dongxing Wang, Chuang Huang, Dongming Wang, Dehui Chang

**Affiliations:** ^1^ Department of Urology, The 940t Hospital of Joint Logistics Support Force of Chinese People’s Liberation Army, Lanzhou, Gansu, China; ^2^ Department of Dermatology, Shuguang Hospital Affiliated to Shanghai University of Traditional Chinese Medicine, Shanghai, China

**Keywords:** extramammary Paget’s disease, immunotherapy, tislelizumab, chemotherapy, surgery

## Abstract

Extramammary Paget disease (EMPD) is a rare epithelial adenocarcinoma in apocrine-gland rich skin, involving the vulva, the scrotum, and the penis. with distant metastases and a poor prognosis. Local EMPD patients generally have a good prognosis, with expected 5-year survival of 60%–92%, but distant metastasis represents poor prognosis and 5-year survival of 10%. Treatment approaches for advanced EMPD are chemotherapy and biological agents, which carry limited efficacy. We report the case of a 57-year-old man diagnosed with metastatic EMPD, who showed a long-term disease control with a combination therapy (an immune checkpoint inhibitor - tislelizumab plus chemotherapy – paclitaxel albumin and cisplatin). This patient underwent a wide penile scrotal lesion excision and six cycles of tislelizumab plus chemotherapy. The patient achieved partial response for the metastatic lesions according to the Response Evaluation Criteria in Solid Tumors (version 1.1). This case report supports further investigation of the combination treatment of chemotherapy and immune checkpoint inhibitors in the management of metastatic EMPD, which currently has an abysmal prognosis and no standardized treatment.

## Introduction

Extramammary Paget disease (EMPD) is an adenocarcinoma originating from the apocrine gland-rich skin (1). EMPD cases are diagnosed mostly as carcinoma *in situ* (2). But, once the EMPD cells invade deep into the dermis, they frequently metastasize to the regional lymph nodes (LNs) and remote organs (3). Metastasis is related with poor prognosis and a 5-year survival rate of <10% due to the limitations of common chemotherapies (4). Although conventional chemotherapies have been used for a long time to treat distant metastases, no prospective study has been conducted showing improved overall survival (OS) with routine chemotherapies (5).

Here, we report a case of metastatic EMPD that responded to the combination treatment of chemotherapy (paclitaxel-albumin and cisplatin) and the immune checkpoint inhibitor tislelizumab.

## Case description

A 57-year-old man presenting scrotal skin ulceration and inguinal lymphadenopathy visited our hospital on December 28, 2022. The patient had no other past history and or similar disease in the family.

**Figure 1:**
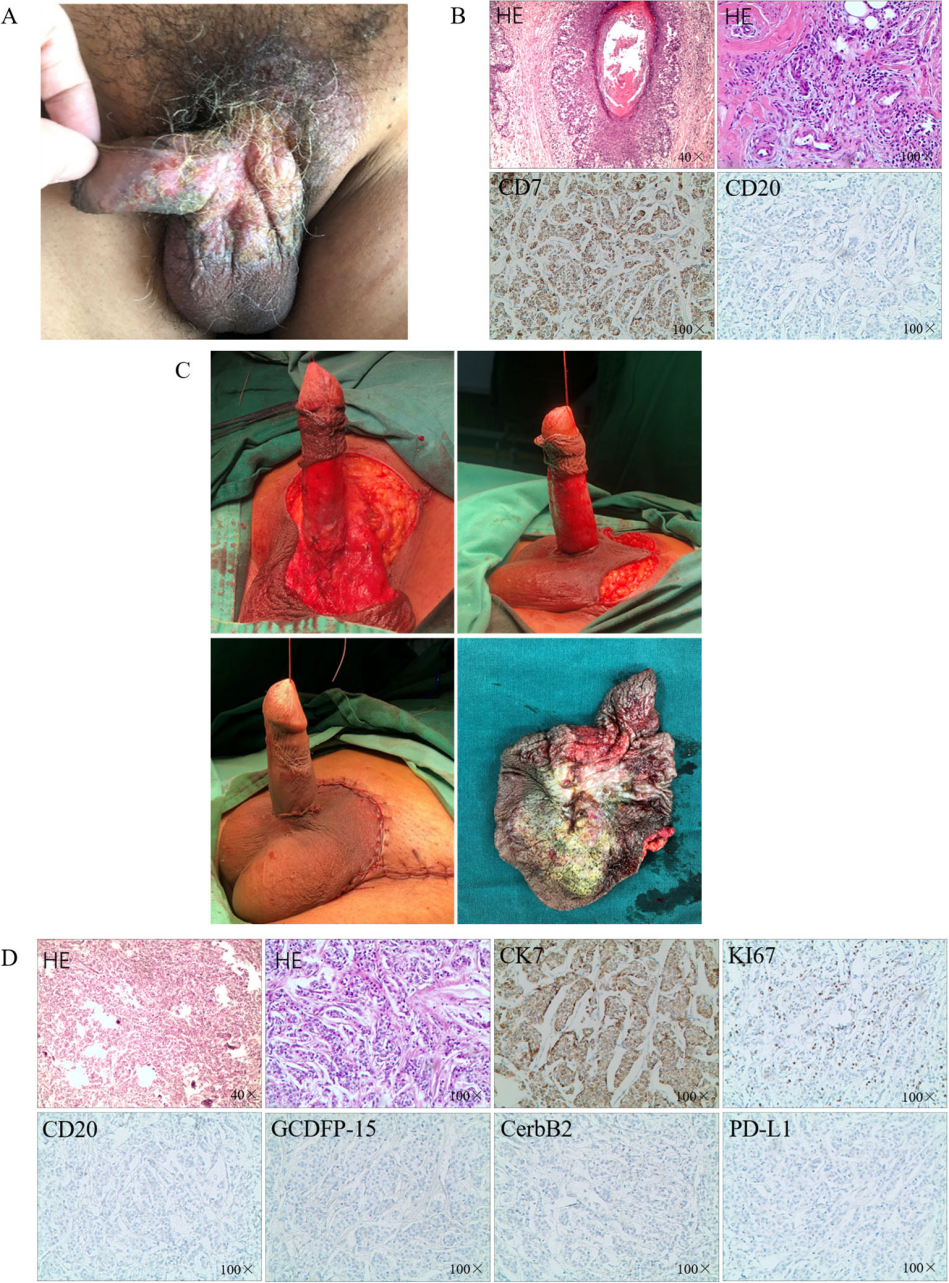
The diagnosis and surgical treatment. **(A)** Erythematous plaque-like lesions on the surface of the penis and scrotum; **(B)** IHC after partial excision; **(C)** The surgery of extended local excision; **(D)** IHC after extended local excision.

Physical examination revealed that the left side of the root of the scrotum and the left side of the root of the penis measured approximately 5 × 5 cm and 3 × 2 cm, respectively, with an eczema-like ulcerated surface. A small amount of purulent secretion was observed on the surface with a faint odor (**Figure 1A**). The left side of the groin had a palpable mass measuring approximately 5 × 3 cm, with poor mobility and no tenderness. The right side of the groin area had a palpable mass measuring approximately 1 cm, with some mobility and no tenderness. There was no obvious abnormality in the external urethral opening.

Laboratory and auxiliary tests were performed. The carcinoembryonic antigen level was 31.42 ng/ml. Chest and whole abdomen computed tomography (CT) showed multiple enlarged and fused LNs in the abdominal cavity, retroperitoneum, and left iliac vessels. The largest one in the left iliac vessels was approximately 4.6 × 8.3 cm, encircling the iliac vessels; several exudate density shadows were seen in the surrounding fat interstitial space.

Based on the clinical presentation and case history, eczema of the scrotum was suspected. We performed partial excision of the perineal skin tumor for histopathological analysis. Carcinoma cells were detected in the epidermis and hair follicles and arranged in nests. These histological features were consistent with those of Paget disease with infiltrating adenocarcinoma. Immunohistochemical staining indicated that the EMPD tumor cells were positive for CK7 and negative for CK20 (**Figure 1B**). Based on the histopathological and immunohistochemical findings, a diagnosis of cT2N1M1 primary EMPD was made (6).

Considering the localized skin breakdown with itching and the patient’s strong desire for surgical treatment of the penile and scrotal lesions, excision of the penile and scrotal lesions including inguinal LN biopsy was planned. Meanwhile, the patient refused stereotactic radiosurgery. The excision area was marked approximately 2 cm along the periphery of the scrotal and penile lesions. The skin layers were separated one by one up to the deep fascial layer, gradually freeing the skin of the lesions toward the center to the root of the penis; no skin lesions were detected at the base. After the scrotal skin was freed, the scrotal wall was lifted upward. The penis was dragged out from the hole in the center of the flap. The circumcision margin was then anastomosed to the scrotal incision, and the scrotal meatus and skin were closed layer by layer (**Figure 1C**). Three left inguinal enlarged LNs were excised and sent for pathologic examination. The histological features were consistent with the previous biopsy findings. Cancer cells were detected in the left inguinal LN. Postoperatively, the pathological diagnosis of the patient was pT2N1M1. Immunohistochemical staining indicated that the EMPD tumor cells were positive for CK7, and Ki-67 (≈20%) and negative for CK20, CerbB-2 and GCDFP-15, along with low expression of PD-L1 (**Figure 1D**).

Systemic therapy is one of the mandatory and effective approaches for patients presenting distant metastases. Considering the limitations of chemotherapy alone, we chose to combine it with immunotherapy. The specific treatment schedules were as follows: Day 1, tislelizumab 200 mg, intravenously (IV); Day 2: paclitaxel-albumin 260 mg/m^2^, IV; Day 3: cisplatin 70 mg/m^2^, IV. This combination treatment was administered as one cycle over a period of 21 days, and the patient received six cycles in total, after which tislelizumab was administered as maintenance therapy for 12 months. During the treatment period, the patient experienced mild nausea and fatigue, and developed grade 2 myelosuppression after the third treatment cycle. After symptomatic treatment, the condition improved, with no other serious adverse reactions observed.

**Figure 2:**
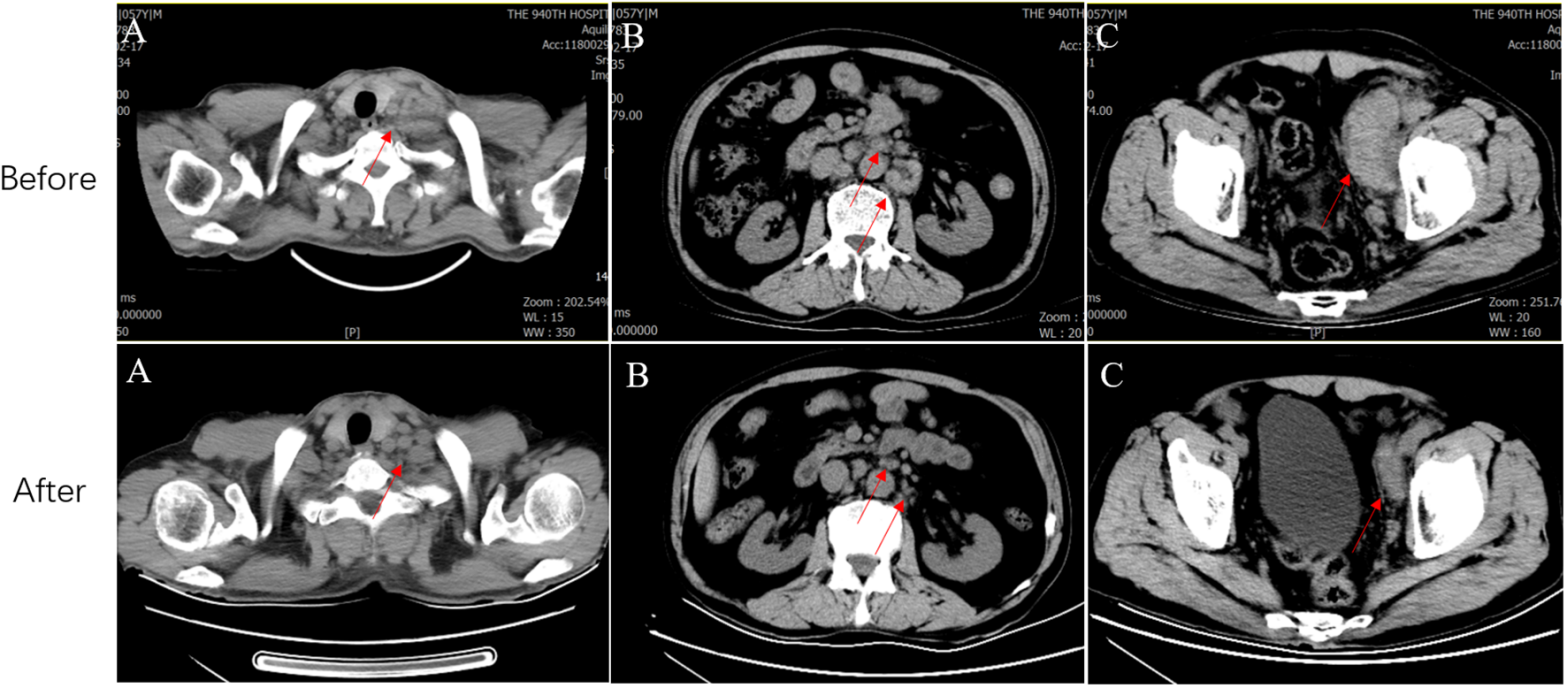
CT before and after systemic therapy. The computed tomography shows multiple enlarged lymph nodes in the neck **(A)**, retroperitoneal **(B)** and pelvic **(C)** regions (Red arrows represent metastatic lymph nodes). CT, Computed tomography.

The patient achieved partial response (PR) for the metastatic lesions according to the Response Evaluation Criteria in Solid Tumors (version 1.1) after six cycles of treatment. CT showed that the cervical, retroperitoneal, and pelvic LNs reduced to varying degrees (**Figure 2**). Compared to the previous CT findings, the cervical, retroperitoneal, and pelvic LNs showed limited changes after six treatment cycles; thus, the disease was considered to have reached a plateau. The patient is undergoing follow-up visits to date.

## Discussion

Chemotherapy remains the mainstream of EMPD management. However, to date, there is no standardized treatment which has been established for distant metastasis (7). Among the various regimens, docetaxel monotherapy and 5-fluorouracil/cisplatin (FP) therapy have been frequently used. Kato et al. analyzed 17 EMPD patients with distant metastases, of which 9 administered best supportive care, whereas 8 were treated with FP (8). The median progression-free survival (mPFS) and median OS (mOS) of the FP group were 6.2 and 19.4 months; the mPFS and mOS of the FP group were not obviously longer than those of the supportive care group (6). Tokuda et al. retrospectively investigated 22 patients with metastatic EMPD who administer FP therapy and indicated that the response rate, mPFS, and mOS were 59%, 5.2 months, and 12 months, respectively (9). Yoshino et al. designed a multicenter retrospective study to estimate the validity of docetaxel as a first-line chemotherapy drug in 13 distant EMPD patients (10). The disease control rate was as high as 83%, and the mPFS, mOS, and 1-year OS were 7.1 months, 16.6 months, and 75.0%, respectively (8). Even though routine chemotherapies have been used to treat metastatic metastases for a long time, the aforementioned studies indicate the possible validity of chemotherapy; however, no prospective study has indicated that routine chemotherapy improves the OS (11). Guercio et al. reported a case of distant EMPD showing treatment response with immune checkpoint inhibitors. The new treatment project, including four cycles of 1 mg/kg ipilimumab plus 3 mg/kg nivolumab, resulted in a long PR lasting 7 months (12). Evidence-based clinical guidelines recommend chemotherapy, or immune checkpoint inhibitors for patients with distant EMPD (5). Based on the aforementioned literature, we aim to investigate whether the combination of immunotherapy and chemotherapy can yield benefits in metastatic Extramammary Paget’s Disease (EMPD).

Another study showed a successful result with immune checkpoint inhibitor therapy in a distant EMPD case, along with low PD-L1 expression (13). Even though EMPD cells commonly were short of better predictors which can reflect tumor response to immune checkpoint inhibitors, such as high PD-L1/L2 expression and microsatellite instability-high status, this does not represent that immune checkpoint inhibitors will elicit a poor anti-tumor effect in all EMPD cases (14). Our case studies also confirm that the combined regimen can achieve favorable therapeutic outcomes in EMPD, even in cases with low PD-L1 expression.

Our study provides preliminary evidence for extending the indications of chemotherapy combined with immunotherapy to metastatic EMPD. The limitation of this study is that it is merely a case report, and further studies of metastatic EMPD cases treated with chemotherapy combined with immunotherapy are warranted to evaluate its efficacy.

## Data Availability

The original contributions presented in the study are included in the article/supplementary material. Further inquiries can be directed to the corresponding author.
